# Determinants of adherence to the Mediterranean diet among adults in Mediterranean countries: a systematic literature review

**DOI:** 10.1017/S1368980025101432

**Published:** 2025-11-07

**Authors:** Cecile Obeid, Anke Oenema, Doris Jaalouk, Stef P.J. Kremers, Jessica S. Gubbels

**Affiliations:** 1 NUTRIM Institute of Nutrition and Translational Research in Metabolism, Department of Health Promotion, Maastricht University, PO Box 616, 6200 MD Maastricht, the Netherlands; 2 Department of Health Psychology, Open Universiteit, PO Box 2960, 6401 DL Heerlen, the Netherlands; 3 College of Arts & Sciences, American University of Iraq Baghdad (AUIB), Baghdad Airport Road, Baghdad, Iraq

**Keywords:** Mediterranean diet, Determinants, Adherence, Mediterranean countries, Adults

## Abstract

**Objective:**

This study aims to provide an overview of evidence on factors affecting Mediterranean diet (MD) adherence across socio-ecological levels (individual, interpersonal and environmental) in Mediterranean countries, which can be target points for future interventions to promote MD adherence.

**Design:**

A systematic review was conducted following the PRISMA (Preferred Reporting Items for Systematic Review and Meta-Analysis) guidelines and registered in the Prospero database (CRD42020189337). Literature was searched in PubMed, Web of Science and PsycINFO.

**Setting:**

The MD is one of the healthiest dietary patterns, reducing risk of chronic disease while promoting better health outcomes. However, adherence to the MD remains challenging, even in Mediterranean countries.

**Participants:**

Healthy adults aged 18 years and older, living in a Mediterranean country.

**Results:**

A total of thirty-seven cross-sectional studies were included, with 190 to 13 262 participants. Most studies (30/37) were conducted in European Mediterranean countries, primarily Italy (*n* 14), Spain (*n* 9) and Greece (*n* 6). All studies involved community-based samples; two studies included only women. Individual-level determinants were the most frequently examined. Higher socio-economic status, regular breakfast consumption, being unemployed, a job seeker or retired were linked to better MD adherence. Socio-cognitive and interpersonal factors were underexplored. At the environmental level, COVID-19 confinement boosted adherence, whereas the effects of economic crises were inconsistent. Effect sizes were mostly very small to small, and findings are based on low-quality studies.

**Conclusions:**

This systematic review highlighted several socio-economic and environmental factors potentially influencing MD adherence. However, more robust research is needed to better understand socio-cognitive and ecological factors.

The Mediterranean diet (MD) is the dietary pattern followed originally by populations of the Mediterranean basin. This dietary pattern has proven to be one of the healthiest and is associated with a reduced risk of a variety of chronic diseases: including CVD^(1,2)^, Alzheimer’s disease, depression^(3–5)^, various types of cancer and cancer-related mortality among the general population and cancer survivors^(6,7)^. The MD has also been found to be associated with better glycemic control, lipid profiles and blood pressure levels, resulting in improved control of cardiovascular risk factors, better management of diabetes type II diabetes and decreased risk of new onset type II diabetes^(8,9)^.

Originally, the MD is characterised by generous consumption of whole grains, vegetables, fruits, seeds, nuts, legumes, olives and olive oil, regular but moderate consumption of dairy products and fish, and very limited intake of processed food, meat and meat products, in addition to moderate wine drinking (with meals)^(1)^. This dietary pattern is characterised by high intake levels of *n*-3 fatty acids, polyphenols, vitamins D and B and complex carbohydrates that play a favourable role in health outcomes^(10)^.

However, despite its well-documented advantages, adherence to the MD is a challenge for many individuals, including those living in Mediterranean countries. A recently published systematic review found that inhabitants of Mediterranean countries show moderate to low adherence to the MD^(11)^. Veronese *et al.* found a notable decline in MD adherence in Italy from 1985–1986 to 2005–2006, especially among younger individuals, mainly due to reduced olive oil consumption^(12)^. The decline in adherence to the MD in Mediterranean countries may indicate that it is important to promote MD adherence in these countries. A starting point for MD adherence promotion is to identify determinants of adherence as this provides valuable input for national nutrition policies of Mediterranean countries^(13)^.

To comprehensively explore the determinants of adherence to the MD, it is crucial to adopt a socio-ecological approach that considers the complex interplay between individual, interpersonal, community and societal determinants^(14)^. The Socio-Ecological Model recognises that individuals exist within multiple layers of influence, from personal factors to broader social, cultural and environmental contexts, which collectively shape their dietary choices and behaviours^(14)^. Several individual sociodemographic characteristics, such as age, gender, occupation and marital status, seem to influence adherence to the MD. In general, older adults are more compliant to the MD, even though there are also mixed findings for the association with age, with some studies indicating a decrease due to barriers like chewing difficulties and financial hardships^(15)^, while others show an increase due to age-related health issues necessitating dietary changes^(16)^. Observed positive associations between older age and MD adherence could further partly reflect period effects, as older generations may have been more exposed to traditional Mediterranean dietary patterns, while younger generations are more influenced by westernised diets^(17)^. Research on sex differences in MD adherence is inconclusive; some studies report better adherence in women then in men^(18)^, while others find no difference^(19)^. In addition, socio-economic factors such as food insecurity may be linked to differences in MD adherence, as indicated by findings from a survey of Lebanese adolescents^(20)^. Socio-cognitive and behavioural factors such as knowledge, attitudes, beliefs, preferences, culinary skills and self-efficacy in meal preparation may also play a crucial role in MD adherence^(21)^.

Moving beyond the individual, interpersonal factors encompass social relationships, support systems and family dynamics that influence dietary choices^(22)^. Family members, friends and colleagues can provide encouragement^(23–25)^ or act as barriers to MD adherence^(26)^. The level of social support, shared meals and cultural norms within these social networks have been found to impact dietary behaviours and shape long-term adherence patterns^(22)^. The physical environment influences MD adherence through factors like availability, accessibility, affordability and proximity to markets and grocery stores that sell the ingredients for a MD and restaurants offering Mediterranean cuisine^(27)^.

Broader political, social and economic environmental factors can also influence adherence to the MD. Societal factors such as cultural norms, economic conditions, food policies and marketing strategies also shape dietary patterns^(21,28)^. Social norms, educational initiatives and cultural traditions emphasising fresh, healthy foods support MD adherence, while economic disparities and food marketing can either facilitate or hinder it^(29,30)^. Research indicates that socio-economic factors are major drivers in the shift away from the MD towards Western diets and convenience foods, although some studies found no significant association^(31)^.

However, while several factors have been implicated as influencing adherence to the MD, there is currently no comprehensive overview of the determinants affecting adherence within Mediterranean countries. This gap in the literature highlights the need for an integrated analysis of determinants across multiple socio-ecological levels. Therefore, this study aims to provide an overview of the existing evidence on determinants of MD adherence, categorised into three socio-ecological levels: individual, interpersonal and environmental, encompassing community, organisational and policy influences^(32)^.

## Methodology

A systematic review of studies reporting on the determinants of adherence to the MD among adults from Mediterranean countries was conducted as per PRISMA (Preferred Reporting Items for Systematic Review and Meta-Analysis) 2020 guidelines^(33)^. The review protocol was registered in the Prospero database under the registration number: CRD42020189337. The initially approved protocol was modified by splitting the review of the literature into two sub-studies: one focused on the level of adherence to the MD in Mediterranean countries (published elsewhere^(11)^) and the current review focused on determinants of adherence. In addition, we have changed the quality assessment tool, shifting from the Newcastle–Ottawa Scale (NOS) to the National Collaborating Center for Methods and Tools (NCCMT) tool^(34)^, which is better suited to the study designs included in our review. No other major modifications were made.

### Selection criteria for studies

The inclusion criteria were the following: any type of quantitative studies conducted among adults (mean age above 18 years in the studied sample), living in a Mediterranean country (i.e. Albania, Algeria, Bosnia, Croatia, Cyprus, Egypt, France, Gibraltar, Greece, Israel, Italy, Lebanon, Libya, Morocco, Malta, Monaco, Montenegro, Palestinian territory, Slovenia, Spain, Syria, Turkey and Tunisia) and using a validated dietary assessment and scoring tool to quantify adherence to the MD (e.g. the Greek Mediterranean Index (MedDietScore)^(35)^ or the Mediterranean Diet Scale (MDS)^(36)^). Also, studies should investigate determinants affecting adherence to the MD whether on the individual, interpersonal and/or environmental level and should present a quantitative statistical analysis of the association between the determinant and MD adherence. Studies that solely included populations with chronic illnesses, co-morbidities or a high risk of nutrition-related disorders (e.g. inflammatory bowel diseases, CVD, diabetes, kidney diseases or wasting diseases such as cancer and HIV), or with a condition that affects the ability to independently choose food intake (e.g. documented dementia, Alzheimer’s disease or psychological disorders such as schizophrenia) were excluded. We also excluded studies among specific subpopulations such as pregnant women, centenarians or athletes, to focus on the general population. In addition, we excluded studies that assessed *only* sociodemographic factors (e.g. age, gender, marital status and education) and/or health behaviours (e.g. smoking and physical activity) in relation to MD adherence, as these are often considered *correlates* rather than primary *determinants*. We aimed to focus on broader-level influences, including environmental, social and policy-related factors, to provide more original insights.

### Literature search

In order to perform a comprehensive search of the literature, three databases were searched from the inception of the database until September 2024: PubMed, PsycINFO and Web of Science. The search strategy was formed by a combination of controlled descriptors (indexers in each database) and keywords, according to the indication offered in each electronic database. The search strategy encompasses keywords for both exposures and outcomes, including determinants across socio-ecological levels (individual, interpersonal and environmental factors) as well as descriptors of adherence to the MD among adults in Mediterranean countries. This comprehensive approach thoroughly captures of relevant studies for the research topic. The search strategies for all the databases can be found in Appendix S1.

**Figure 1.**
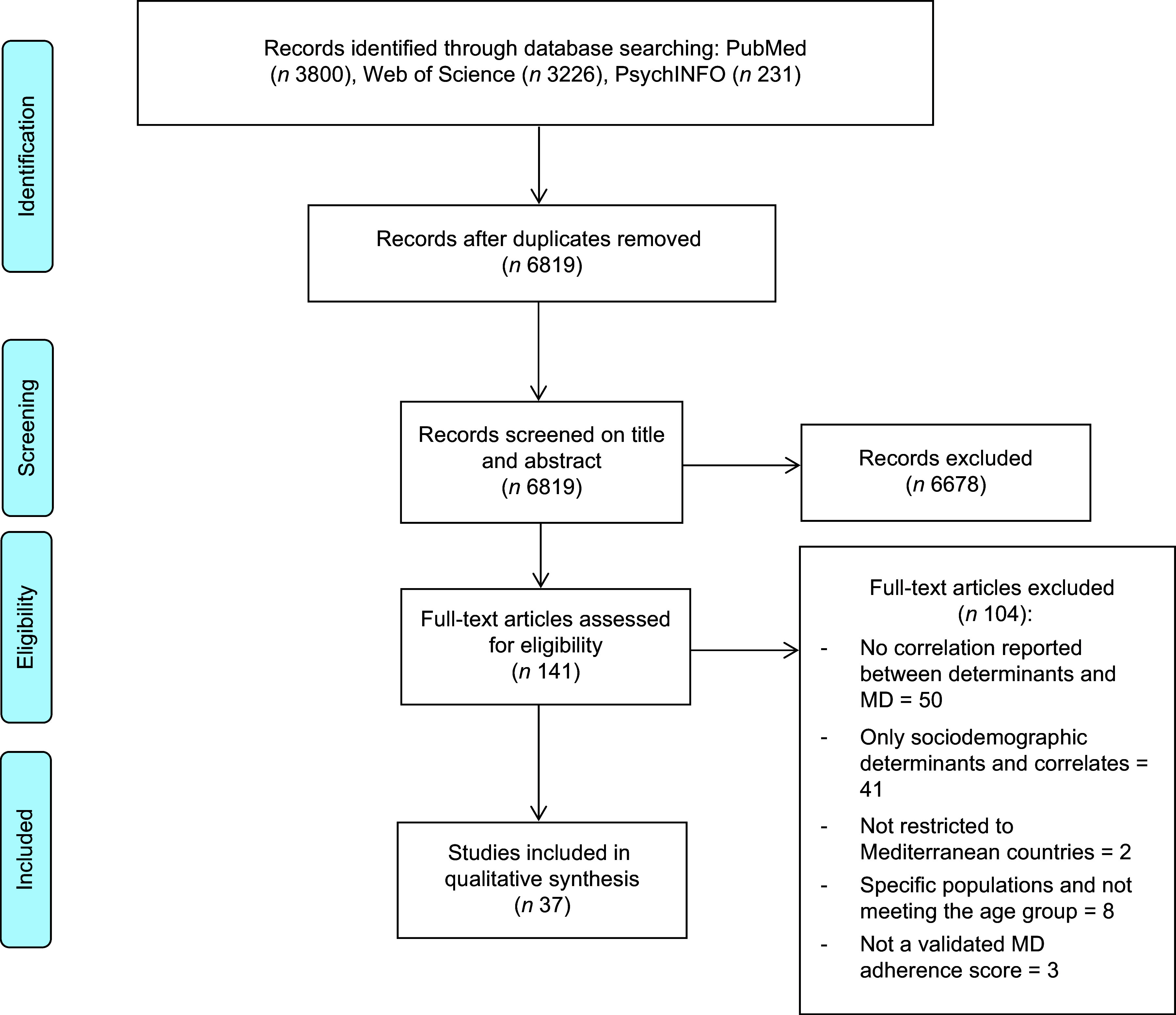
Flow chart result of the search strategy. MD, Mediterranean diet.

After identifying the records, the selection process was done using the Rayyan Qatar Computing Research Institute (QCRI) website^(37)^. After removing duplicates by CO, articles were screened against the inclusion and exclusion criteria first on the title and then on the abstract by CO as well. Excluded articles were then verified by a second author (DJ), and discrepancies were resolved by discussion and confirmed with two other authors (JG and AO). Then, the full texts were individually screened for final selection by CO and DJ (see flow chart in Figure 1).

### Data extraction and quality assessment

Included articles were independently assessed by both assessors (CO and NA), and data were extracted for each included article and tabulated in an Excel file. Disagreements were resolved through consensus with a third person (JG/AO). The following data were extracted from the articles: name of first author and year of publication, study design, date of data collection, general population’s characteristics (nationality, sample size, age, sex, education level, socio-economic status, marital status, urban/rural living and BMI), the score used to assess MD adherence and, if reported, the mean of MD adherence in the general population. In addition, we extracted the types of determinants of MD adherence, the statistical analyses used and the corresponding results. For studies reporting several statistical models, we prioritised those adjusted for covariates, using the model identified by the authors as their main or primary estimate. If this was not specified, we selected the most comprehensively adjusted model (Table 1). Studies were categorised according to the extent of covariate adjustment. We defined well-adjusted studies as those that accounted for a comprehensive set of relevant covariates, including key demographic, behavioural and clinical factors. Moderately well-adjusted studies included some, but not all, of these covariates, typically adjusting for a limited subset such as age and sex but omitting other important confounders. Finally, poorly adjusted or unadjusted studies either controlled for very few covariates or provided no adjustment at all, thereby increasing the risk of residual confounding (Table 2).

**Table 1.** Overview of study design, sample size, sample characteristics and determinants of MD adherence of the included studies

	Reference	Study design (name of cohort)	Country	Date of data collection	Sample size	Sample characteristics (sociodemographic)* by order: mean age, age range, gender distribution, education, geographical distribution, marital status, income and employment status	Determinants (specific constructs)	Adjustment for potential confounders	MD adherence score	Mean (sd) MD adherence score
1	Alvarez-Fernandez C et al. (2021)^(38)^	CS	Spain	2016–2017	1609	Local government workers: 48 % (8·3), 66·8 % males workers at risk of social exclusion: 41·1 % (10·2), 23·1 % males	SES	Not adjusted	MEDAS	7·7 (2·0)
2	Benedetti I et al. (2016)^(39)^	CS	Italy	1997–2012	NR	46·98; > 14; 48·1 % males; years of education: 41·66 % > 8; 30·43 % married, 55·26 % unmarried, 14·31 % divorced or widowed	Income level, economic crisis	SD; lifestyle factors	MDS	NR
3	Bonaccio M et al. (2012)^(40)^	CS	Italy	2005–2010	13 262	53 (11); > 35; 50 % males	SES	SD; Eating habits; lifestyle factors	MDS	4·44 (1·64)
4	Turki et al. (2022)^(41)^	CS	Tunisia	2021	1082	32·5 (12); 20–74; 25·7 % males; 91·8 % university graduates	SES	SD; Health status	MEDAS	6·6 (1·07)
5	Mamalaki E et al. (2019)^(25)^	CS	Greece	2011–2014	1993	73 (6); 65–99; 41 % males; years of education 7·7(4·8)	Economic classes	SD	MedDietScore	33·3 (4·6)
6	Moreno-Agostino C et al. (2018)^(42)^	CS	Spain	2014–2015	2397	61·87 (15·2); 21–101; 46·1 % males; 85·65 % urban	Job-related factors and social factors such as living alone, number of social contacts per month	SD; lifestyle; health status	MEDAS	8·55 (1·95)
7	Yassibas E et al. (2023)^(43)^	CS	Turkey	NR	1732	27·7(10); 35·5 % males; 1·7 % primary school level 2 % secondary school level 12 % high school level 78·1 % university level 6·2 % postgraduate level	Health consciousness, education, age	SD	MEDAS	6·92 (2·07)
8	Biasini B et al. (2021)^(44)^	CS	Italy	2019	838	18–65; 48 % males; 7·4 % primary or lower secondary, 53·5 % secondary, 39·1 % tertiary; northwest 26·3 %, northeast 20 %, central 19·9 %, south 22·9 %, islands 10·9 %	Sustainable nutrition knowledge, and environmentally responsible food choices score	Not adjusted	MDS	4·0 (3·0–5·0)
9	La Fauci V et al. (2020)^(45)^	CS	Italy	2019	507	17–35; 24 % males; 0·6 % middle school 83 % upper secondary school 16·4 % university graduates; 75·7 % living with their families, 17·6 % lived with other people 6·3 % living alone	Eating habits, self-perceived image	Not adjusted	KIDMED	NR
10	Zappala G et al. (2019)^(46)^	CS	Italy	2014–2015	1952	> 18**	Eating habits: eating Breakfast, fruits and veggies intake and place of eating	SD; lifestyle factors	Medi-Lite	NR
11	Lotti et al. (2022)^(47)^	CS	Italy	2021–2022	1247	36·1; 33·3 % males; 54·2 % have a university degree; 63·4 % single or unmarried	Eating habits	Not adjusted	Medi-Lite	9·7 (2·2)
12	Gonzalez Pascual J et al. (2024)^(48)^	CS	Spain	2019	775	22·6 (1·5); 30·4 % males	Gender typologies: feminine, masculine, androgynous, undifferentiated	SD; health status	MEDAS	6·4
13	Uliano A et al. (2024)^(49)^	CS	Italy	NR	190	30·47 (9·98); 49·47 % males; 4·21 % secondary school 36·84 % high school 58·95 % university degree or higher; 26·32 % suburb area 16·32 % rural area 57·37 % urban area.	Sustainable nutrition knowledge, and environmentally responsible food choices	Not adjusted	Medi-Lite	NR
14	Rodríguez-Muñoz et al. (2020)^(50)^	CS	Spain	2017–2018	464	20·93 (3·28); 33 % males; 93·4 % undergraduate students; 96·1 % single; 44·6 % cohabited with their parents/tutors 43·1 % with other students in rental apartments	Having a morning chronotype	SD; health behaviour	KIDMED	NR
15	Benedetti I et al. (2018)^(51)^	CS	Italy	1997–2012	NR	> 14**	Chronotype (morning/evening), sexual opinion	SD	MDI	NR
16	Zaragoza-Marti A et al. (2018)^(52)^	CS	Spain	NR	351	71·06; > 60; 42·77 % males mean years of education: 8·73 (5·59); 79·5 % urban	Eating habits, number of household components	SD; lifestyle behaviour; health status	MDS	4·93 (1·58)
17	Godos J et al. (2019)^(53)^	CS	Italy	2014–2015	2044	48·1 (17·5); > 18	Life satisfaction	SD; lifestyle factors; health status	Medi-Lite	NR
18	Laiou E et al. (2020)^(24)^	CS CREDITS4HEALTH (C4H)	Italy, Spain and Greece	2015	1572	42 (32–50); 18–65; 39·4 % males; 35·4 %university, 31·0 % high school, 7·9 %primary/middle school, 25·7 % missing; 32·2 % single or divorced 41·5 % married or cohabiting 0·9 % widow/widower 25·4 % missing	Quality of life	Not adjusted	MEDAS	7 (6–9)
19	Marfil-Carmona R et al. (2021)^(28)^	CS	Spain	NR	634	35·18 (9·68) 18–66 44·5 % males	Availability of social and emotional support	Not adjusted	KIDMED	NR
20	Bonaccio M et al. (2017)^(3)^	CS	Italy	2005–2010	1829	28–83; 49·7 % males 19·3 % postgraduate or higher, 35·8 % upper secondary, 44·9 % up to lower secondary; 46·9 % northern, 15·1 % central or 38 % southern; 10·1 % single, 78·8 % married, 4·6 % divorced, 6·5 % widow	Economic crisis	Not adjusted	MedDietScore	27 (4·5)
21	Kritsotakis G et al. (2014)^(54)^	CS	Greece	2007	377	29·6 (5); 100 % females; education: 16·8 % low, 46·3 % medium, 37 % high; 79·1 % urban; 89·5 % married/engaged, 10·5 % other	Self-reported negative impact of the economic crisis on diet	Not adjusted	MDS	NR
22	Apostolaki I et al. (2021)^(55)^	CS	Greece	2014–2015	436	75 (6·2); > 65; 57·6 % males education: 6 (5–6) years 75·7 % married	Social capital	SD; health status	MDS	32 (3·6)
23	Bonanni A et al. (2013)^(56)^	CS	Italy	2009–2010	883	52·5 (10); > 35; 50·1 % males; education: 48·2 % low, 51·8 % high	Social capital, reading food labels	SD; lifestyle factors	MDS	Read food labels no: 4·2 (1·6)/yes: 4·5 (1·6)
24	Bonaccio M et al. (2013)^(57)^	CS	Italy	2009–2010	744	52·1 (9·4); > 35; 50·3 % males; > 8 years of education 52 %; 85·2 % married/ living-in partners	Reading food labels	SD; lifestyle factors	MDS	Low nutrition knowledge: 4·19 (1·64)/ medium: 4·44 (1·61)/ high: 4·55 (1·56)
25	Bonaccio M et al. (2011)^(58)^	CS	Italy	NR	959	53 (10); > 35; 50 % males	Psychological well-being, mass media pressure	SD; lifestyle factors; health status	MDS	4·37 (1·61)
26	Sánchez-Villegas A et al. (2002)^(59)^	CS	Spain	2000	3847	39·9 years males and 35·3 years females; 41·2 % males	Nutrition knowledge (Self-awareness of HDL-cholesterol levels)	SD; lifestyle behaviours; medical history	MDP	NR
27	Scali J et al. (2000)^(60)^	CS	Southern France	1994–1996	964	20–76; 49 % males; 16 % primary school, 43·1 % high school 40·9 % university	Living in rural areas	SD; lifestyle factors	MDQI	NR
28	Katsarou A et al. (2010)^(61)^	CS	Cyprus	NR	300	75 (7·1); 65–100; 38 % males; 31 % living alone; 39 % rural	Place of residence	SD; lifestyle factors	MDS	33·5 (3·4)
29	Tsiampalis TH et al. (2020)^(27)^	CS	Greece	2001–2012	2749	38·69 (1·51); 48·27 % males; % of green urban areas 1·70	Living alone, cohabiting, geographical location	Not adjusted	MDS	71·74 (7·88)
30	Esin K. et al. (2024)^(62)^	CS	Turkey	NR	2039	20·9 (1·5)	Food insecurity (FI)	Not adjusted	MEDAS	Food Secure: 6 (2·02) Food Insecure: 5·3 (1·82)
31	Sahin G. A et al. (2024)^(63)^	CS	Turkey	2022–2023	2675	29·5 (10·35); 18–49; 100 % females; 5·4 % primary school 3·4 % middle school 17·2 % high school 68·6 % university level 5·3 % master’s degree/doctorate; 60·3 % unmarried; 66·3 % not working.	Income, FI	Not adjusted	MEDAS	5·8 (2·23)
32	Franco E et al. (2021)^(64)^	CS (Healthy Cities by Sanitas)	Spain	2020	297	42·76 (7·79); 24–63 50·2 % males	Household food insecurity; number of main meals and snacks	Not adjusted	PREDIMED	NR
33	Shyam S et al. (2023)^(65)^	CS	Spain	2013–2023	1065	38·7 (12·4); > 16; 27·2 % males; 84·3 % urban; 9·7 % living alone, 20·9 % couple, 5·1 % with children and without partner, 39·3 % couple and children, 21·3 % parents, 2·4 % couple, children and parents, 1·3 % parents and children	Confinement due to COVID-19 pandemic	SD; lifestyle factors health status	PREDIMED	NR
34	Atabilen et al. (2024)^(66)^	CS	Turkey	2020–2021	6609	18–70; 30 % males; > 70 % university graduates	Confinement due to COVID-19 pandemic	Not adjusted	MEDAS	NR
35	Pavlidou E et al. (2024)^(67)^	CS	Greece	2018–2023	3388	74·54 (8·4); 49·2 % males; 29 % primary education, 31·6 % secondary education 39·4 % university level; 84·7 % unemployed; 28·2 % rural areas	COVID-19 Phobia	SD; lifestyle factors; socio-cognitive status	MedDietScore	28·4 (4·7) during pre-COVID-19 period
36	Scoditti E et al. (2024)^(68)^	CS	Italy	2022	748	40–59; 42·4 % males; 82·1 % graduated or with a higher level of education	COVID-19 period	SD; SES; socio-cognitive status	MEDAS	During work from home: 7·31 (1·75) Before work from home: 6·66 (1·88)
37	Pavlidou E et al. (2023)^(69)^	CS	Greece	2020–2021	3721	37·6 (5·8); 21–65; 49·5 % males; mean value of the educational years 12·2 (2·8) (range: 0–14 years); 71·6 % low yearly income; 73·1 % lived with others 26·9 % lived alone; 80·8 % employed; 74·9 % urban regions	Family financial level	SD; anthropometric characteristics; lifestyle	MedDietScore	27·5 (4·8)

MD, Mediterranean diet; CS, cross-sectional; SES, socio-economic status; MEDAS, Mediterranean Diet Adherence Screener; MDI, Mediterranean Diet Index; MDP, Mediterranean Diet Pattern; MDQI, Mediterranean Diet Quality Index; PREDIMED, Prevention with Mediterranean Diet; KIDMED, Mediterranean Diet Quality Index for children and adolescents; NR, not reported; SD, sociodemographic; MDS, Mediterranean Diet Scale.

SD variables include age, gender, education, income, employment status, geographical distribution and marital status. MDS is by Trichoupoulo et al. MedDietScore is by Panagiotakos et al. MEDAS is by Schroder et al. Medi-Lite score is by Sofi et al.

*The data (on the total general population) that was presented in the studies is reported.

**No background characteristics reported on the general population but rather stratified by group of MD adherence.

**Table 2.** Overview of the number of studies investigating determinants of adherence to the MD clustered by Socio-Ecological Model levels (individual, interpersonal and environmental), including the direction of associations and the adjustment for covariates (numbers refer to the reference number)

Determinants of MD adherence	Number of studies	Statistically significant (+) association	No statistically significant association	Statistically significant (–) association
Individual				
Socio-economic factors				
Income-related factors (income, wealth status and having financial support)	7	(47)^*^_VS_, (42)^§^_M_, (40)^*^_S_, (66)^§^_S_, (74)^*^_S_	(24)^§^	(39)^†^_S_
SES	4	(54)^a^, (71)^*^_M_, (70)^*^_L_	(40)^*^	
Unemployed (housewives) and job seekers	2	(39)^†^_M_		(55)^*^_M_
Being retired	2	(55)^*^_S_, (39)^†^_VS_		
Independent/living alone, number of household components.	5	(24)^§^_S_	(47)^*^, (71)^*^, (42)^§^, (25)^†^	
Having one household member < 18 *v*. none or two	1	(42)^§^_M_		
Socio-cognitive determinants				
Exhibiting an androgynous typology *v*. feminine, masculine or undifferentiated typology	1	(46)^*^_M_		
Health and nutrition knowledge (including reading food labels)	4	(58)^*^_L_, (51)^*^_S_, (50)^*^_S_, (41)^§^_S_		
Considering MD a sustainable dietary model	2	(42)^§^_M_	(65)^‡^	
Behaviour and eating habits				
Frequency of snacking and having sweet snacks	2			(43)^§^_L_, (44)^*^_L_
Having a morning chronotype	2	(45)^§^_S_, (56)^b ‡^_L_		
Eating breakfast	3	(43)^§^_L_, (47)^*^_M_		(44)^*^_M_
Higher number of meals and snacks	1	(66)^§^_S_		
Having lunch or dinner as the main meal *v*. breakfast as a main meal.	1	(47)^*^_S_		
Healthy eating habits: fruits, vegetables and water.	2	(43)^§^_L_, (47)^*^_VS_		
Frequency of eating out	4		(44)^*^	(47)^*^_S_, (42)^§^_S_, (43)^§^_VL_
Responsibility of food purchase and preparation	1		(42)^§^	
Taking part in solidarity purchasing groups or environmental associations	1		(42)^§^	
Self-perceived adoption of a sustainable dietary model	1	(42)^§^_L_		
Quality of life				
Psychological well-being	1	(28)^§^_M_		
Mental component quality of life	1		(62)^*^	
Social capital	2	(62)^*^_S_	(61)^§^	
Physical component quality of life	1	(62)^*^_S_		
Quality of life (high *v*. other quality of life)	1	(48)^*^_L_		
Fairly satisfied and not very satisfied *v*. very satisfied with leisure time	2	(47)^*^_VS_	(25)^†^	
Fairly, not very, not at all satisfied with one’s economic situation *v*. very satisfied	1	(47)^*^_S_		
Other categories *v*. very satisfied with family relationships or with friendships	1			(47)^*^_S_
Interpersonal (socio-cultural)				
Emotional support availability, number of close friends, number of social contacts per months	2	(24)^§^_S_, (25)^†^_VS_		
Number of intellectual activities	1	(25)^†^_VS_		
Attending church or religious services	1		(24)^§^	
Motor, physical, management, interpersonal dimension of the job, complexity dimension of the job	1		(25)^†^	
Tolerance of diversity	1	(61)^§^_S_		
Environmental				
Mass media pressure (MMP)	2	(53)^*^_S_		(28)^§^_S_
Confinement due to COVID-19 pandemic	5	(59)^§^_L_, (60)^*^_VS_, (68)^§^_S_, (72)^*^_M_, (52)^*^_M_		
Economic crisis	2		(39)^†^	(49)^a§^
Food insecurity	2		(66)^§^	(67)^§^_S_
Living in rural areas	2	(71)^*^_M_, (69)^‡^_VS_		
Geographical area of residence, municipality’s sector	2	(27)^§^_S_	(42)^§^	
Percent green area	1	(27)^**c**§^_S_	(27)^**d**§^	
Number of street markets and supermarkets	1	(27)^§^_S_ for street markets VS for supermarkets		
Percent immigrants, percent unemployment rate	1			(27)^§^_S_ for immigrants and M for unemployment

MD, Mediterranean diet; SES, socio-economic status.

Abbreviations of classification of effect sizes: VS, very small, S, small, M, medium, L, large.

a Standardised effect sizes could not be calculated for this study as required information was not presented in the article. ^b^They found a significant negative association between Evening Chronotype and adherence to MD and results were reversed to align with the coding in the other study; ^c^for moderate and high SES areas; ^d^ for low SES areas.

* Studies that adjusted well for covariates.

† Studies that adjusted moderately well for covariates.

‡ Studies that poorly adjusted for covariates.

§ Studies that did not adjust for covariates.

Quality assessment was conducted using the NCCMT scale for assessment of quantitative studies^(34)^. The NCCMT tool incorporates seven aspects of study quality: selection bias, study design, confounders, blinding, data collection methods, withdrawals and drop-outs and intervention integrity. To make the tool suitable for assessment of observational studies, the seventh aspect in the tool (intervention integrity) was replaced with an assessment of the validity and reliability of the tools used to assess the determinants. The quality assessment was performed by CO, JG and AO. To ensure consistency in scoring, inter-rater reliability was assessed by having all three reviewers independently evaluate 35 % of the papers (nine studies). Any disagreements in their assessments were discussed and resolved through consensus, establishing a standardised approach to scoring. Using this agreed-upon framework, the remaining studies were divided and evaluated independently by the three reviewers (CO, JG and AO), ensuring that the same criteria were applied uniformly across all studies.

### Data analysis

The studies were initially grouped based on the type of determinants they examined and mapped to the three condensed levels of the Socio-Ecological Model^(14)^, which include individual factors, interpersonal factors and environmental determinants (social, physical and economic). Within each determinant, findings were further categorised into three groups based on the reported relationship with adherence to the MD in each study: significant positive association, no significant association and significant negative association. These categorisations were systematically organised in Table 2, showing the direction and significance of the relationship between the determinant and MD adherence reported in the individual studies. If a study reported determinants at multiple levels, it was included in each of the relevant clusters accordingly (Table 2). To facilitate interpretation and allow comparison across studies, we added an indication of effect size to Table 2. Reported effect sizes for OR, *β* coefficients and correlation coefficients were classified as small, medium or large according to Cohen’s guidelines^(37)^. When studies reported unstandardised regression coefficients, we calculated standardised coefficients by multiplying the unstandardised coefficient by the ratio of the standard deviation of the independent variable to that of the dependent variable, ensuring consistency in effect size interpretation.

### Synthesis of the evidence

We used a qualitative approach to data synthesis, by counting the number of studies that investigated a specific determinant and calculating the percentage of studies that found an association in the same direction. If two-thirds or more of the studies found an association in the same direction, we considered this as evidence of an association, given that three or more studies examined the same association. When only two studies examined the same association, we considered this as a potential association. Determinants that were only investigated in one study were not interpreted.

## Results

### Study selection

The search of the three databases yielded a total of 7257 records (Figure 1). After removing duplicates (number of deleted records *n* 438), 6819 unique articles remained. During the screening of articles’ titles and abstracts, 6678 were excluded. The number of retained articles for full-text screening was 141. In total, 104 articles were excluded after full-text screening. Reasons to exclude articles were the paper not being limited to Mediterranean countries, being focused on specific subpopulations (e.g. athletes, health majors’ students and pregnant females), not within the defined age range (≥ 18 years), studies assessing only sociodemographic determinants (e.g. age, gender, marital status and educational level) and/or health correlates (e.g. smoking and physical activity), the study did not report a statistical analysis of associations between determinants and MD adherence or they did not use a validated MD adherence score. The final number of included studies was 37. The result of the selection procedure is summarised in Figure 1 ^(70)^.

### Characteristics of included studies

Fourteen studies were conducted in Italy^(39,40,44–49,51,53,56–58,68,71)^, nine in Spain^(28,38,42,48,50,52,59,64,65)^, six in Greece^(25,27,54,55,72,73)^, four in Turkey^(43,62,63,66)^, one across Italy, Spain and Greece^(24)^ and one from each of the following countries France^(60)^, Tunisia^(41)^ and Cyprus^(61)^ (Table 1). All thirty-seven studies had a cross-sectional design. The number of participants in the included studies ranged between 190 and 13 262. Most studies were performed on a community-based sample and presented data for both males and females. Two studies included women only^(54,63)^. Various MD scores were used. The MDS was used the most (*n* 11)^(27,39,40,44,52,54–58,61)^ followed by ten papers that used the fourteen-item Mediterranean Diet Adherence Screener (MEDAS)^(24,38,41–43,48,62,63,66,68)^, four each used the MediLite score^(46,47,49,53)^ and the MedDietScore^(25,67,71,72)^, three used the Mediterranean Diet Quality Index for children and adolescents score^(28,45,50)^, two used the Prevention with Mediterranean Diet score^(64,65)^ and one each used the Mediterranean Diet Index^(51)^, Mediterranean Dietary Pattern^(59)^ or the Mediterranean Diet Quality Index^(60)^ scores. Of the thirty-seven included studies, twenty-three adjusted for covariates, while fourteen did not. Among those that adjusted, fourteen were well adjusted, six were moderately well adjusted and three were poorly adjusted. As summarised in Table 2, clearer and more consistent associations with MD adherence were generally reported in well-adjusted studies (e.g. those on health and nutrition knowledge or COVID-19 confinement). By contrast, studies with limited or no covariate adjustment more often yielded weaker or inconsistent associations. Effect sizes across the included studies were mostly very small to small, with occasional medium and large effects. Socio-economic factors and behavioural determinants typically showed very small to small associations with MD adherence. Health and nutrition knowledge presented stronger effects, ranging up to large. Quality of life, socio-cultural and environmental factors were generally associated with small effects, though COVID-19 confinement showed larger effects in some cases. Standardised effect sizes could not be calculated for several studies due to insufficient data (Table 2).

### Quality analysis of the included studies

The quality assessment of the included studies, conducted using the NCCMT scale^(34)^, revealed that thirty-six out of thirty-seven studies received an overall poor-quality score, with only one study achieving a fair rating. Common issues leading to low scores included poor rating for the section selection bias due to unrepresentative samples, inadequate reporting of participant agreement rates, as well as limited documentation of the reliability of data collection and exposure assessment tools. Withdrawals and drop-out rates were often poorly reported, with only two studies meeting the criteria for a good score. Further details on the quality assessment are provided in online supplementary material, Supplemental Information 2 (S2).

### Determinants of Mediterranean diet adherence

On the individual level, the financial status of the individual assessed through income, wealth status or financial support were investigated in seven studies, with five^(40,44,51,67,74)^ showing a statistically significant positive association, one^(24)^ indicating no significant association and one^(39)^ showing a negative association with MD adherence. Socio-economic status (SES) was examined in four studies, with three^(38,41,61)^ indicating a positive association and one^(40)^ showing no significant association with MD adherence. The impact of living alone or having fewer household members was explored in five studies, with one^(24)^ showing a positive association in ameliorating the adherence to MD, while the others^(25,44,51,61)^ found no significant association. Health and nutrition knowledge, including reading food labels, consistently showed a positive association with adherence in all four^(49,56,57,59)^ studies where it was investigated. Regular breakfast consumption was also examined in three studies, with two^(45,51)^ showing a positive association and one^(46)^ a negative association. Conversely, the frequency of eating out, investigated in four studies, showed a negative association in three^(44,45,51)^ studies, while one^(46)^ study found no significant association with adherence.

Other individual-level determinants were examined in two studies. Being unemployed or a job seeker and being retired^(39,42)^ both showed a positive association with adherence in both studies where they were examined. Having a morning chronotype^(47,50)^ demonstrated a positive association in the two studies that investigated it. In addition, although the consumption of fruits, vegetables and water^(45,51)^ was reported to have a positive association with MD adherence in two of two studies, these components are integral parts of most MD scores. Therefore, findings related to their isolated consumption should be interpreted with caution, as they likely reflect associations intrinsic to the scoring methodology rather than representing independent determinants of adherence. Social capital (assessed as part of quality of life) was investigated in two studies, with one^(55)^ showing a positive association and the other^(54)^ showing no significant association. Considering MD as a sustainable diet showed a significant positive association in one study^(44)^ and no significant association in the other^(43)^. Lastly, the degree of satisfaction with leisure time was explored in two studies, with one^(51)^ finding a positive association and the other^(25)^ finding no significant association.

On the interpersonal level, the availability of emotional support, the number of close friends and number of social contacts per months were investigated in two^(24,25)^ studies, both showing a significant positive association with adherence to the MD.

On the environmental level, the impact of the confinement due to COVID-19 was examined in five^(64–66,68,69)^ studies with all of them showing a significant positive association, indicating that confinement was associated with more MD adherence. Living in rural areas was positively associated with adherence in the two studies^(60,61)^ where it was examined. The geographical area of residence or the municipality’s sector was investigated in two studies, with one^(27)^ showing a positive association and the other^(44)^ showing no significant association. The impact of the economic crisis^(39,71)^ was investigated in two studies with one showing a significant negative association and one showing no significant association. This pattern was also found for the impact of food insecurity^(62,63)^ where one study found a positive and one a negative association with MD adherence.

## Discussion

This systematic review aimed to provide a comprehensive overview of the evidence on determinants of adherence to MD among adults across Mediterranean countries. Individual-level determinants were the most frequently examined in the included studies, with less focus on interpersonal and environmental factors. Among the individual-level determinants, socio-economic factors were most frequently explored. Notably, at the environmental level, an improvement in adherence to the MD during the COVID-19 confinement was observed, while broader environmental factors such as mass media pressure, place of living and economic crisis remained underexplored. The search strategy identified thirty-seven articles that fulfilled the inclusion criteria, mostly reporting determinants of MD adherence from European Mediterranean countries, except for one study from Tunisia. The findings highlight a significant gap in research from Middle Eastern and North African Mediterranean countries, indicating that the existing evidence is still limited. Although a wide range of determinants were identified, their associations with adherence to the MD were generally modest, highlighting the multifactorial but subtle influence of individual, socio-cultural and environmental factors.

Even though individual-level determinants were the most frequently examined in the studies, the variety of factors examined was limited and mostly related to socio-economic factors, while only a few studies examined socio-cognitive determinants. Socio-economic factors like income, wealth status and financial support showed a positive association with MD adherence. This aligns with previous research that links financial stability to better access to healthy food^(63,69)^ and that higher SES often results in better health knowledge and dietary habits^(38)^. Notably, unemployment and retirement also showed a positive association in the current review, although these might be associated with a lower income and SES. This might indicate different underlying mechanisms, potentially because unemployment and retirement allow more meal preparation time^(35,36)^.

Our findings also highlighted that health and nutrition knowledge showed a positive association with MD adherence, as did eating breakfast regularly. These findings suggest that individuals who better understand of nutrition principles tend to align their dietary choices more closely with the MD. Studies have found that those with better knowledge of nutrition show significantly better adherence to the MD, including regular consumption of vegetables, fruits and other key food items recommended in the MD^(75,76)^. While regular breakfast consumption was associated with greater adherence to the MD, this relationship may reflect an overall healthier eating pattern rather than breakfast itself being a causal factor. Individuals who consistently eat breakfast tend to engage in dietary behaviours more aligned with MD recommendations throughout the day, regardless of the distribution of food intake^(77)^. The results suggest that targeting nutrition knowledge and habitual practices, such as eating breakfast, may serve as potential strategies for promoting adherence to the MD.

This review did not identify studies addressing socio-cognitive determinants, such as attitudes, beliefs, social pressures and confidence in adopting the MD. This is an omission, since understanding socio-cognitive determinants is essential for developing tailored interventions that address the psychological and social factors influencing dietary behaviours and long-term adherence to the MD. Therefore, future research focusing on these factors is needed to provide a deeper understanding of individual determinants of MD adherence beyond socio-economic influences^(78)^. Qualitative studies have pointed towards nutritional attitudes and beliefs, perceived positive outcomes and confidence as potential facilitators to MD adherence^(79)^, indicating the need to confirm these findings in quantitative studies.

Eating out frequently showed evidence of a potential association in this review, where eating out less frequently is positively associated with MD adherence. Frequent eating out has been associated in the literature with poorer adherence to the MD due to the higher intake of unhealthy foods, which are more common in restaurants compared to home-cooked food^(45)^. The ability to plan and cook meals at home supports healthier eating patterns, reinforcing that time available for meal preparation plays a critical role in adopting the MD^(80)^.

Interpersonal determinants of adherence to the MD have not been extensively examined in the literature. Only emotional support and social connections showed a potential positive association with MD adherence in both studies that were included. Emotional support from family and friends, along with social connections, boosts long-term dietary adherence by providing motivation and accountability^(81)^. While interpersonal factors are underexplored, these results underscore the potential importance of supportive social networks in sustaining the MD. More research is needed to understand the impact of such connections on dietary choices, especially that sharing meals is a core principle of the MD^(80)^.

Environmental determinants of MD adherence also remain underexplored. A notable factor is the impact of COVID-19 confinement, which showed evidence of a positive association with MD adherence. The positive change in dietary habits during COVID-19 confinement can be explained by having more time at home for meal preparation and fewer opportunities to dine out^(68,82)^. The impact of economic crisis and food insecurity on MD adherence in this systematic review showed mixed results. While two studies found a significant negative association, supporting the theory that financial hardship leads to reduced access to fresh, healthy foods and lower MD adherence^(62,71)^, two others did not find a significant association. While some previous research suggests that economic crises may, in specific contexts, promote better adherence to traditional diets^(83)^, the findings of this review highlight the variability in outcomes and the potential for economic hardship to also negatively affect MD adherence. Further research is needed to investigate the role of food availability, accessibility and affordability in MD adherence, as these factors were not explored in the included studies. Promoting local food practices and enhancing government support programmes could be potential strategies to address these gaps in future interventions. Living in rural areas showed a potential positive association with MD adherence in our review. This can perhaps be explained by rural residents often having better access to fresh, local produce and traditional foods, which align with MD principles. Additionally, rural lifestyles may favor healthier, unprocessed foods over the convenient and fast-food options which are more common in urban areas as seen in previous research^(61,71)^. This finding highlights a key opportunity for future policy, where interventions could be more targeted at urban populations. Policies that focus on improving access to fresh produce, encouraging local food markets and promoting cooking skills might help urban populations adopt healthier eating patterns similar to those in rural areas. Moreover, there is a lack of studies addressing the influence of mass media pressure, including food marketing strategies and media promotion, on adherence to the MD.

The quality assessment of the included studies indicated significant methodological limitations, with 36 out of 37 studies rated as poor overall and only one study achieved a fair score. Major issues included unrepresentative sampling, inadequate reporting on participant agreement rates and insufficient documentation of the reliability of data collection and exposure assessment tools. Reporting withdrawals and drop-out rates was also often inadequate, further limiting the reliability of findings. These limitations underscore the need for higher-quality research to provide more robust evidence.

This systematic review provides some indication of potential targets for interventions. It can guide the development of policies to promote sustained adherence to this beneficial dietary pattern, while also highlighting gaps in the current evidence on determinants of MD adherence across different socio-ecological levels. However, the included studies were all cross-sectional and of low quality, limiting the ability to directly inform intervention design directly. More well-designed research is needed, particularly in Middle Eastern and North African Mediterranean countries, to better understand these determinants and support the development of effective, evidence-based strategies. Several potentially important factors influencing adherence to the MD have not yet been thoroughly studied or consistently replicated. The included studies primarily focused on individual-level socio-economic determinants, with comparatively less attention given to interpersonal and environmental factors. This indicates the need for a broader research scope of research to delve into the multiple layers of determinants affecting MD adherence. Moreover, as shown in Table 2, variation in the extent of covariate adjustment represents a methodological limitation: studies with comprehensive adjustment more often yielded clear and directionally consistent associations with MD adherence, whereas poorly adjusted or unadjusted analyses produced weaker or contradictory findings. This pattern suggests that the heterogeneity across studies may partly reflect differences in analytical rigour, underscoring the need for standardised and transparent covariate adjustment in future research.

This systematic review has several strengths: it followed the PRISMA guidelines and was preregistered in the Prospero database (CRD42020189337). Multiple databases were searched: PubMed, Web of Science and PsycINFO, in order to collect a broad range of articles from different research fields. In addition, the screening, data extraction and quality assessment were performed by independent researchers, and the tool used to assess the quality of the included studies was a validated instrument^(34)^.

Several limitations should be acknowledged in this systematic literature review. First, the search was limited to studies published in English, which may have excluded relevant research published in other languages, particularly from Middle Eastern Mediterranean countries where studies may be reported in Arabic or French. However, during the screening process, only a small number of non-English articles were identified and excluded, suggesting that the impact of this language restriction on the comprehensiveness of our review is likely minimal. Nevertheless, we acknowledge this as a potential limitation, as it may have led to the omission of context-specific evidence from non-English sources. Second, our review focused on the general population and excluded specific subgroups such as pregnant women, centenarians and athletes, as these groups may have distinct motivations for adhering to the MD. While we may have missed potentially relevant determinants applicable to the general population, only three studies were excluded for this reason, and we do not expect their omission to have substantially altered our findings. Third, to minimise selection bias and enhance the comprehensiveness and validity of our results, we systematically included both exposures and outcomes in the search terms. To further ensure the robustness of our search strategy, we validated it by testing for the inclusion of key articles known to be relevant to our research question. This validation increased our confidence that the likelihood of missing important studies is limited. However, as with any systematic review, it remains possible that some relevant studies were not identified, which is an inherent limitation. Fourth, for data organisation, some determinants with nuanced differences in meaning were grouped into broader categories, which may have led to a loss of granularity when interpreting context-specific factors. Additionally, we did not interpret determinants that were investigated in only one study, as isolated findings lack sufficient evidence for generalisation. While these decisions helped streamline the synthesis and highlight consistent patterns, they may have resulted in the underrepresentation of unique or less commonly studied determinants. Fifth, given the heterogeneity of the included studies, we employed a qualitative approach for data analysis. Although the method of counting the number of studies reporting associations in the same direction is widely used, it remains a crude approach that does not account for the strength of associations, differences in study quality, sample size or statistical power. Despite these limitations, this synthesis provides valuable insights into which categories of determinants have been most frequently explored and, perhaps more importantly, highlights gaps in the existing evidence base. Sixth, while we extracted the most advanced or fully adjusted models as reported by study authors, we acknowledge the potential for overadjustment bias in some cases, particularly where mediators or colliders may have been included. In the absence of explicit causal frameworks in most studies, distinguishing between confounders and post-exposure variables was not always feasible, which may have influenced the estimated associations.

Additional limitations stem from the scientific evidence itself. First, all included studies were cross-sectional, and many determinants lacked replicated findings. Furthermore, there was considerable variability in the extent and reporting of covariate adjustment. While most studies accounted for key demographic variables such as age and sex, the inclusion of other covariates – such as socio-economic indicators, lifestyle factors or clinical characteristics – varied, reflecting differences in study objectives and analytical strategies rather than a uniform set of confounders. Moreover, several studies did not clearly report which covariates were adjusted for, limiting the ability to assess the robustness of their findings. This variability in adjustment strategies may introduce bias and should be considered when interpreting the overall results. Lastly, most of the included studies were conducted in European countries, which may limit the generalisability of our findings to other regions, particularly the Middle East and North Africa, where cultural and economic contexts differ. This highlights the need for further research on the determinants of MD adherence in these underrepresented regions.

## Conclusion

This systematic review aimed to provide an overview of determinants potentially associated with MD adherence across socio-ecological levels. Socio-economic factors (income, wealth status, financial support, unemployment and retirement) and health and nutrition knowledge were consistently associated with better adherence, along with regular breakfast consumption, as derived from cross-sectional studies. This review found no studies investigating attitudes, beliefs, confidence in adopting the MD, or the role of emotional support and social connections, highlighting the need for further research on these potential determinants. Environmental factors, such as the positive impact of COVID-19 confinement, and the mixed effects of economic crisis and food insecurity, also warrant deeper exploration.

While the findings are inconclusive, they suggest potentially relevant target points for intervention. These include improving socio-economic conditions and implementing health education programmes to increase nutrition knowledge. Strengthening social support networks and addressing challenges like food insecurity may also play a role in supporting adherence to the MD. Further research is needed to confirm these as effective intervention strategies. Future research should focus on investigating socio-cognitive determinants, the interpersonal and environmental factors related to MD adherence, in well-designed studies, to determine the key target points for comprehensive strategies for long-term adherence to the MD among adults in Mediterranean countries.

## Supporting information

Obeid et al. supplementary material 1 Obeid et al. supplementary material

Obeid et al. supplementary material 2 Obeid et al. supplementary material

## Supplementary material

The supplementary material for this article can be found at https://doi.org/10.1017/S1368980025101432.
