# Supplementary material for: Determinants of adherence to the Mediterranean diet among adults in Mediterranean countries: a systematic literature review
Source: Public Health Nutr. 2025 Nov 7;28(1):e194. doi: 10.1017/S1368980025101432 (PMC12722083; doi:10.1017/S1368980025101432)
Supplement: Obeid et al. supplementary material 1 — Obeid et al. supplementary material [file S1368980025101432sup001.docx]

***Appendix S2:* Table of quality analysis of included studies**

|  | **Authors** | **A Section Selection bias Rating** | **B design global rating** | **C Section Selection bias Rating** | **D section Blinding rating** | **E Section Data collection methods Rating** | **F Section Withdrawals and drop-outs Rating** | **G exposure assessment rating** | **Global rating** |
| --- | --- | --- | --- | --- | --- | --- | --- | --- | --- |
| 1 | Alvarez-Fernandez C (2021) | 1 good | 3 poor | 3 Poor | 2 Fair | 2 Fair | not applicable | 3 Poor | 3 poor (two or more poor ratings) |
| 2 | Benedetti I (2016) | 3 poor | 3 poor | 2 fair | 3 Poor | 2 fair | 3. poor (two or more poor ratings) | 3 Poor | 3 poor (two or more poor ratings) |
| 3 | Bonaccio M (2012) | 2 Fair | 3 poor | 1 good | 3 Poor | 2 Fair | not applicable | 3 Poor | 3 poor (two or more poor ratings) |
| 4 | Turki et al. (2022) | 2 Fair | 3 poor | 1 good | 3 Poor | 2 fair | not applicable | 3 Poor | 3 poor (two or more poor ratings) |
| 5 | Mamalaki E (2019) | 3 Poor | 3 poor | 2 fair | 2 Fair | 2 Fair | not applicable | 2 Fair | 3 poor (two or more poor ratings) |
| 6 | Moreno-Agostino, C (2018) | 2 Fair | 3 poor | 1 good | 2 Fair | 2 Fair | 2. fair (one poor rating) | 1 Good | 2 fair (one poor rating) |
| 7 | yassibas E et al (2023) | 3 poor | 3 poor | 2 fair | 3 Poor | 2 Fair | 3. poor (two or more poor ratings) | 3 poor | 3 poor (two or more poor ratings) |
| 8 | Biasini B (2021) | 2 Fair | 3 poor | 1 good | 3 Poor | 2 fair | not applicable | 3 Poor | 3 poor (two or more poor ratings) |
| 9 | La Fauci V (2020) | 3 Poor | 3 poor | 3 Poor | 2 Fair | 2 Fair | not applicable | 1 Good | 3 poor (two or more poor ratings) |
| 10 | Zappala G (2019) | 2 Fair | 3 poor | 1 good | 3 Poor | 2 fair | 3. poor (two or more poor ratings) | 3 Poor | 3 poor (two or more poor ratings) |
| 11 | Lotti et al. (2022) | 3 poor | 3 poor | 3 Poor | 3 Poor | 2 Fair | not applicable | 2 Fair | 3 poor (two or more poor ratings) |
| 12 | Gonzalez Pascual J et al (2024) | 3 poor | 3 poor | 2 fair | 3 Poor | 2 fair | not applicable | 1 Good | 3 poor (two or more poor ratings) |
| 13 | Uliano A et al. (2024) | 3 poor | 3 poor | 3 Poor | 3 Poor | 2 fair | 3. poor (two or more poor ratings) | 2 fair | 3 poor (two or more poor ratings) |
| 14 | Rodríguez-Muñoz et al 2020 | 3 poor | 3 poor | 2 fair | 3 Poor | 2 Fair | not applicable | 2 Fair | 3 poor (two or more poor ratings) |
| 15 | Benedetti I (2018) | 2 Fair | 3 poor | 2 fair | 1 good | 2 Fair | not applicable | 3 Poor | 3 poor (two or more poor ratings) |
| 16 | A. ZARAGOZA-MARTÍ (2018) | 3 Poor | 3 poor | 2 fair | 3 Poor | 2 Fair | not applicable | 2 Fair | 3 poor (two or more poor ratings) |
| 17 | Godos J (2019) | 1 good | 3 poor | 1 good | 2 Fair | 2 Fair | 1. good (no poor ratings) | 1 Good | 3 poor (two or more poor ratings) |
| 18 | Laiou E (2020) | 3 Poor | 3 poor | 3 Poor | 2 Fair | 2 Fair | not applicable | 2 Fair | 3 poor (two or more poor ratings) |
| 19 | Marfil-Carmona R (2021) | 3 Poor | 3 poor | 3 Poor | 2 Fair | 2 Fair | 2. fair (one poor rating) | 1 Good | 3 poor (two or more poor ratings) |
| 20 | Bonaccio M (2017) | 3 Poor | 3 poor | 3 Poor | 3 Poor | 3 Poor | not applicable | 3 Poor | 3 poor (two or more poor ratings) |
| 21 | Kritsotakis G (2014) | 2 Fair | 3 poor | 3 Poor | 3 Poor | 2 Fair | not applicable | 2 Fair | 3 poor (two or more poor ratings) |
| 22 | Apostolaki I (2021) | 3 poor | 3 poor | 1 good | 3 Poor | 2 Fair | not applicable | 2 Fair | 3 poor (two or more poor ratings) |
| 23 | Bonanni A (2013) | 2 Fair | 3 poor | 1 good | 3 Poor | 2 Fair | not applicable | 1 Good | 3 poor (two or more poor ratings) |
| 24 | Bonaccio M (2013) | 2 Fair | 3 poor | 1 good | 3 Poor | 2 Fair | not applicable | 2 Fair | 3 poor (two or more poor ratings) |
| 25 | Bonaccio M (2011) | 2 Fair | 3 poor | 1 good | 3 Poor | 2 Fair | not applicable | 2 Fair | 3 poor (two or more poor ratings) |
| 26 | A. Sánchez-Villegas (2002) | 3 Poor | 3 poor | 1 good | 2 Fair | 2 Fair | not applicable | 3 Poor | 3 poor (two or more poor ratings) |
| 27 | Scali J (2000) | 3 Poor | 3 poor | 1 good | 3 Poor | 2 Fair | not applicable | 3 Poor | 3 poor (two or more poor ratings) |
| 28 | Katsarou A (2010) | 2 Fair | 3 poor | 1 good | 3 Poor | 2 fair | not applicable | 3 Poor | 3 poor (two or more poor ratings) |
| 29 | Tsiampalis TH (2020) | 2 Fair | 3 poor | 3 Poor | 3 Poor | 2 fair | 1. good (no poor ratings) | 2 Fair | 3 poor (two or more poor ratings) |
| 30 | Esin K et al (2024) | 2 Fair | 3 poor | 3 Poor | 3 poor | 2 Fair | not applicable | 1 good | 3 poor (two or more poor ratings) |
| 31 | Sahin G. A et al (2024) | 3 poor | 3 poor | 3 Poor | 2 fair | 2 Fair | not applicable | 1 good | 3 poor (two or more poor ratings) |
| 32 | Franco E (2021) | 3 Poor | 3 poor | 3 Poor | 3 Poor | 2 Fair | 3. poor (two or more poor ratings) | 1 Good | 3 poor (two or more poor ratings) |
| 33 | shyam S (2023) | 1 good | 3 poor | 1 good | 2 Fair | 2 Fair | not applicable | 3 Poor | 3 poor (two or more poor ratings) |
| 34 | Atabilen et al. (2024) | 3 poor | 3 poor | 3 Poor | 3 poor | 2 Fair | not applicable | 1 good | 3 poor (two or more poor ratings) |
| 35 | Pavlidou E et al (2024) | 3 poor | 3 poor | 2 fair | 3 poor | 2 Fair | not applicable | 1 good | 3 poor (two or more poor ratings) |
| 36 | Scoditti E et al (2024) | 1 good | 3 poor | 2 fair | 3 poor | 2 Fair | not applicable | 1 good | 3 poor (two or more poor ratings) |
| 37 | Pavlidou E et al (2023) | 3 poor | 3 poor | 2 fair | 3 poor | 2 Fair | not applicable | 1 good | 3 poor (two or more poor ratings) |
