# Supplementary material for: Determinants of adherence to the Mediterranean diet among adults in Mediterranean countries: a systematic literature review
Source: Public Health Nutr. 2025 Nov 7;28(1):e194. doi: 10.1017/S1368980025101432 (PMC12722083; doi:10.1017/S1368980025101432)
Supplement: Obeid et al. supplementary material 2 — Obeid et al. supplementary material [file S1368980025101432sup002.docx]

| Database | Search strategy* |
| --- | --- |
| Medline/ PubMed  *September 2024* ^a^ | #1 ((Mediterranean diet[MeSHb Terms] OR "Mediterranean diet"[Title/Abstract] OR "Mediterranean dietary pattern"[Title/Abstract]))  #2 (("Determinant"[Title/Abstract] OR "Determinants"[Title/Abstract] OR "cause"[Title/Abstract] OR "causes"[Title/Abstract] OR "factor"[Title/Abstract] OR "factors"[Title/Abstract] OR "correlat*"[Title/Abstract] OR "predict*"[Title/Abstract] OR "relat*"[Title/Abstract] OR "associat*"[Title/Abstract] OR "influence"[Title/Abstract] OR "influences"[Title/Abstract] OR "influencing"[Title/Abstract] OR "influenced"[Title/Abstract] OR "effect"[Title/Abstract] OR "effects"[Title/Abstract] OR "income"[Title/Abstract] OR "education*"[Title/Abstract] OR "occupation*"[Title/Abstract] OR "religio*"[Title/Abstract] OR "employment"[Title/Abstract] OR "socio-economic"[Title/Abstract] OR "socioeconomic"[Title/Abstract] OR "socio-demographic"[Title/Abstract] OR "sociodemographic"[Title/Abstract] OR "marital status"[Title/Abstract] OR "beliefs"[Title/Abstract] OR "belief"[Title/Abstract] OR "attitude"[Title/Abstract] OR "attitudes"[Title/Abstract] OR "cognition*"[Title/Abstract] OR "Knowledge"[Title/Abstract] OR "norm intention*"[Title/Abstract] OR "taste"[Title/Abstract] OR "habit*"[Title/Abstract] OR "family*"[Title/Abstract] OR "friend"[Title/Abstract] OR "friends"[Title/Abstract] OR "social network*"[Title/Abstract] OR "peer*"[Title/Abstract] OR "social support"[Title/Abstract] OR "social pressure"[Title/Abstract] OR "culture"[Title/Abstract] OR "tradition*"[Title/Abstract] OR "sociocultural"[Title/Abstract] OR "socio-cultural"[Title/Abstract] OR "Home"[Title/Abstract] OR "household"[Title/Abstract] OR "community"[Title/Abstract] OR "neighborhood*"[Title/Abstract] OR "workplace"[Title/Abstract] OR "school*"[Title/Abstract] OR "food retail*"[Title/Abstract] OR "food service outlet*"[Title/Abstract] OR "restaurant"[Title/Abstract] OR "grocery store"[Title/Abstract] OR "supermarket*"[Title/Abstract] OR "shops"[Title/Abstract] OR "university*"[Title/Abstract] OR "work"[Title/Abstract] OR "job"[Title/Abstract] OR "incentive*"[Title/Abstract] OR "social influence"[Title/Abstract] OR "generation*"[Title/Abstract] OR "guideline*"[Title/Abstract] OR "recommendation*"[Title/Abstract] OR "media"[Title/Abstract] OR "food label*"[Title/Abstract] OR "advert*"[Title/Abstract] OR "promotion*"[Title/Abstract] OR "food environment"[Title/Abstract] OR "commercials"[Title/Abstract] OR "marketing"[Title/Abstract] OR "availability"[Title/Abstract] OR "accessibility"[Title/Abstract] OR "Household income"[Title/Abstract] OR "food import"[Title/Abstract] OR "pricing policies"[Title/Abstract] OR "food cost"[Title/Abstract] OR "food costs"[Title/Abstract] OR "consumer*"[Title/Abstract] OR "food insecurity"[Title/Abstract] OR "food security"[Title/Abstract] OR "polic*"[Title/Abstract] OR "polit*"[Title/Abstract] OR "law"[Title/Abstract] OR "regulation*"[Title/Abstract] OR "legislation*"[Title/Abstract] OR "rule*"[Title/Abstract]))  #3 #1 AND #2 |
| PsychINFO^f^  *September 2024* | **#1** (TI^c^ Mediterranean diet OR AB^d^ Mediterranean diet OR MA Mediterranean diet OR TI Mediterranean dietary pattern OR AB Mediterranean dietary pattern)  **#2** (("Determinant"[Title/Abstract] OR "Determinants"[Title/Abstract] OR "cause"[Title/Abstract] OR "causes"[Title/Abstract] OR "factor"[Title/Abstract] OR "factors"[Title/Abstract] OR "correlat*"[Title/Abstract] OR "predict*"[Title/Abstract] OR "relat*"[Title/Abstract] OR "associat*"[Title/Abstract] OR "influence"[Title/Abstract] OR "influences"[Title/Abstract] OR "influencing"[Title/Abstract] OR "influenced"[Title/Abstract] OR "effect"[Title/Abstract] OR "effects"[Title/Abstract] OR "income"[Title/Abstract] OR "education*"[Title/Abstract] OR "occupation*"[Title/Abstract] OR "religio*"[Title/Abstract] OR "employment"[Title/Abstract] OR "socio-economic"[Title/Abstract] OR "socioeconomic"[Title/Abstract] OR "socio-demographic"[Title/Abstract] OR "sociodemographic"[Title/Abstract] OR "marital status"[Title/Abstract] OR "beliefs"[Title/Abstract] OR "belief"[Title/Abstract] OR "attitude"[Title/Abstract] OR "attitudes"[Title/Abstract] OR "cognition*"[Title/Abstract] OR "Knowledge"[Title/Abstract] OR "norm intention*"[Title/Abstract] OR "taste"[Title/Abstract] OR "habit*"[Title/Abstract] OR "family*"[Title/Abstract] OR "friend"[Title/Abstract] OR "friends"[Title/Abstract] OR "social network*"[Title/Abstract] OR "  peer*"[Title/Abstract] OR "social support"[Title/Abstract] OR "social pressure"[Title/Abstract] OR "culture"[Title/Abstract] OR "tradition*"[Title/Abstract] OR "sociocultural"[Title/Abstract] OR "socio-cultural"[Title/Abstract] OR "Home"[Title/Abstract] OR "household"[Title/Abstract] OR "community"[Title/Abstract] OR "neighborhood*"[Title/Abstract] OR "workplace"[Title/Abstract] OR "school*"[Title/Abstract] OR "food retail*"[Title/Abstract] OR "food service outlet*"[Title/Abstract] OR "restaurant"[Title/Abstract] OR "grocery store"[Title/Abstract] OR "supermarket*"[Title/Abstract] OR "shops"[Title/Abstract] OR "university*"[Title/Abstract] OR "work"[Title/Abstract] OR "job"[Title/Abstract] OR "incentive*"[Title/Abstract] OR "social influence"[Title/Abstract] OR "generation*"[Title/Abstract] OR "guideline*"[Title/Abstract] OR "recommendation*"[Title/Abstract] OR "media"[Title/Abstract] OR "food label*"[Title/Abstract] OR "advert*"[Title/Abstract] OR "promotion*"[Title/Abstract] OR "food environment"[Title/Abstract] OR "commercials"[Title/Abstract] OR "marketing"[Title/Abstract] OR "availability"[Title/Abstract] OR "accessibility"[Title/Abstract] OR "Household income"[Title/Abstract] OR "food import"[Title/Abstract] OR "pricing policies"[Title/Abstract] OR "food cost"[Title/Abstract] OR "food costs"[Title/Abstract] OR "consumer*"[Title/Abstract] OR  "food insecurity"[Title/Abstract] OR "food security"[Title/Abstract] OR "polic*"[Title/Abstract] OR "polit*"[Title/Abstract] OR "law"[Title/Abstract] OR "regulation*"[Title/Abstract] OR "legislation*"[Title/Abstract] OR "rule*"[Title/Abstract]))  **#3** #1 AND #2 |
| Web of Science  *September 2024* | **#1** (TS=Mediterranean diet OR "Mediterranean dietary pattern")  **#2** (("Determinant"[Title/Abstract] OR "Determinants"[Title/Abstract] OR "cause"[Title/Abstract] OR "causes"[Title/Abstract] OR "factor"[Title/Abstract] OR "factors"[Title/Abstract] OR "correlat*"[Title/Abstract] OR "predict*"[Title/Abstract] OR "relat*"[Title/Abstract] OR "associat*"[Title/Abstract] OR "influence"[Title/Abstract] OR "influences"[Title/Abstract] OR "influencing"[Title/Abstract] OR "influenced"[Title/Abstract] OR "effect"[Title/Abstract] OR "effects"[Title/Abstract] OR "income"[Title/Abstract] OR "education*"[Title/Abstract] OR "occupation*"[Title/Abstract] OR "religio*"[Title/Abstract] OR "employment"[Title/Abstract] OR "socio-economic"[Title/Abstract] OR "socioeconomic"[Title/Abstract] OR "socio-demographic"[Title/Abstract] OR "sociodemographic"[Title/Abstract] OR "marital status"[Title/Abstract] OR "beliefs"[Title/Abstract] OR "belief"[Title/Abstract] OR "attitude"[Title/Abstract] OR "attitudes"[Title/Abstract] OR "cognition*"[Title/Abstract] OR "Knowledge"[Title/Abstract] OR "norm intention*"[Title/Abstract] OR "taste"[Title/Abstract] OR "habit*"[Title/Abstract] OR "family*"[Title/Abstract] OR "friend"[Title/Abstract] OR "friends"[Title/Abstract] OR "social network*"[Title/Abstract] OR "  peer*"[Title/Abstract] OR "social support"[Title/Abstract] OR "social pressure"[Title/Abstract] OR "culture"[Title/Abstract] OR "tradition*"[Title/Abstract] OR "sociocultural"[Title/Abstract] OR "socio-cultural"[Title/Abstract] OR "Home"[Title/Abstract] OR "household"[Title/Abstract] OR "community"[Title/Abstract] OR "neighborhood*"[Title/Abstract] OR "workplace"[Title/Abstract] OR "school*"[Title/Abstract] OR "food retail*"[Title/Abstract] OR "food service outlet*"[Title/Abstract] OR "restaurant"[Title/Abstract] OR "grocery store"[Title/Abstract] OR "supermarket*"[Title/Abstract] OR "shops"[Title/Abstract] OR "university*"[Title/Abstract] OR "work"[Title/Abstract] OR "job"[Title/Abstract] OR "incentive*"[Title/Abstract] OR "social influence"[Title/Abstract] OR "generation*"[Title/Abstract] OR "guideline*"[Title/Abstract] OR "recommendation*"[Title/Abstract] OR "media"[Title/Abstract] OR "food label*"[Title/Abstract] OR "advert*"[Title/Abstract] OR "promotion*"[Title/Abstract] OR "food environment"[Title/Abstract] OR "commercials"[Title/Abstract] OR "marketing"[Title/Abstract] OR "availability"[Title/Abstract] OR "accessibility"[Title/Abstract] OR "Household income"[Title/Abstract] OR "food import"[Title/Abstract] OR "pricing policies"[Title/Abstract] OR "food cost"[Title/Abstract] OR "food costs"[Title/Abstract] OR "consumer*"[Title/Abstract] OR  "food insecurity"[Title/Abstract] OR "food security"[Title/Abstract] OR "polic*"[Title/Abstract] OR "polit*"[Title/Abstract] OR "law"[Title/Abstract] OR "regulation*"[Title/Abstract] OR "legislation*"[Title/Abstract] OR "rule*"[Title/Abstract]))  **#3** #1 AND #2 |

^(a)September 2024= Date the search strategy was carried out; (b)MeSH, Medical Subject Headings; (c)TI, Title; (d)AB, Abstract; (e)TS, Topic; (f)PsycINFO, Psychology Information. * Applied filters: English language, age groups: adulthood (18 years and older).^
